# Rehabilitation robotics: pilot trial of a spatial extension for MIT-Manus

**DOI:** 10.1186/1743-0003-1-5

**Published:** 2004-10-26

**Authors:** Hermano I Krebs, Mark Ferraro, Stephen P Buerger, Miranda J Newbery, Antonio Makiyama, Michael Sandmann, Daniel Lynch, Bruce T Volpe, Neville Hogan

**Affiliations:** 1 Massachusetts Institute of Technology, Mechanical Engineering Department, Cambridge, MA, USA; 2 Weill Medical College of Cornell University, Department Neurology and Neuroscience, New York, NY, USA; 3 Burke Medical Research Institute, White Plains, NY, USA; 4 Imperial College, London, UK; 5 Interactive Motion Technologies, Inc., Cambridge, MA, USA; 6 Massachusetts Institute of Technology, Brain and Cognitive Sciences, Cambridge, MA, USA

## Abstract

**Background:**

Previous results with the planar robot MIT-MANUS demonstrated positive benefits in trials with over 250 stroke patients. Consistent with motor learning, the positive effects did not generalize to other muscle groups or limb segments. Therefore we are designing a new class of robots to exercise other muscle groups or limb segments. This paper presents basic engineering aspects of a novel robotic module that extends our approach to anti-gravity movements out of the horizontal plane and a pilot study with 10 outpatients. Patients were trained during the initial six-weeks with the planar module (i.e., performance-based training limited to horizontal movements with gravity compensation). This training was followed by six-weeks of robotic therapy that focused on performing vertical arm movements against gravity. The 12-week protocol includes three one-hour robot therapy sessions per week (total 36 robot treatment sessions).

**Results:**

Pilot study demonstrated that the protocol was safe and well tolerated with no patient presenting any adverse effect. Consistent with our past experience with persons with chronic strokes, there was a statistically significant reduction in tone measurement from admission to discharge of performance-based planar robot therapy and we have not observed increases in muscle tone or spasticity during the anti-gravity training protocol. Pilot results showed also a reduction in shoulder-elbow impairment following planar horizontal training. Furthermore, it suggested an additional reduction in shoulder-elbow impairment following the anti-gravity training.

**Conclusion:**

Our clinical experiments have focused on a fundamental question of whether task specific robotic training influences brain recovery. To date several studies demonstrate that in mature and damaged nervous systems, nurture indeed has an effect on nature. The improved recovery is most pronounced in the trained limb segments. We have now embarked on experiments that test whether we can continue to influence recovery, long after the acute insult, with a novel class of spatial robotic devices. This pilot results support the pursuit of further clinical trials to test efficacy and the pursuit of optimal therapy following brain injury.

## Background

**Figure 1:**
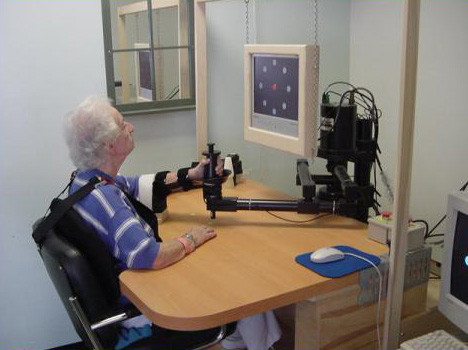
Stroke Inpatient during Therapy at the Burke Rehabilitation Hospital (White Plains, NY). Therapy is being conducted with a commercial version of MIT-MANUS (Interactive Motion Technologies, Inc., Cambridge, MA).

Rather than using robotics as an assistive technology for a disabled individual, our research focus is on the development and application of robotics as a therapy aid, and in particular a tool for therapists. We foresee robots and computers as supporting and enhancing the productivity of clinicians in their efforts to facilitate a disabled individual's functional motor recovery. To that end, we deployed our first robot, MIT-MANUS (see figure 1), at the Burke Rehabilitation Hospital, White Plains, NY in 1994 [1]. In the last ten years, MIT-MANUS class robots have been in daily operation delivering therapy to over 250 stroke patients. Hospitals presently operating one or more MIT-MANUS class robots include Burke (NY), Spaulding (MA), Rhode Island (RI), Osaka Prefectural (Japan) and Helen Hayes (NY) Rehabilitation Hospitals, and the Baltimore (MD) and Cleveland (OH) Veterans Administration Medical Centers.

Most of the work to date has focused on the fundamental question of whether task specific training affects motor outcome and positively influences brain recovery. These efforts directly confront the overwhelming task of reversing the effects of natural injury where lesion size, type and location profoundly determine outcome, and applying controlled conditions in environment and training – nurture – to exploit the ability of the mature nervous system to learn, adapt and change.

Through our work with MIT-MANUS, providing task specific training for patients' with moderate to severe hemiparesis, we have gathered convincing evidence (summarized below) that nurture has a significant impact in speeding motor recovery of the paretic shoulder and elbow, and that robot therapy is effective in delivering the necessary exercise. This recovery is most pronounced in the trained muscle groups and limb segments. Encouraged by these positive results, we have expanded our project to develop a family of novel, modular robots, designed to be used independently or together to rehabilitate other muscle groups and limb segments. This paper describes two different implementations of a new module developed at MIT that expands the capabilities of MIT-MANUS to include motion in a three-dimensional workspace. We will present both implementations, the basic engineering differences between these modules, and pilot clinical results from their use with stroke patients.

### Proof-of-Concept

Volpe [2] reported the composite results of robotic training with 96 consecutive stroke inpatients admitted to Burke who met inclusion criteria and consented to participate [3-7,2]. Patients were randomly assigned to either an experimental or control group and although the patient groups were comparable on all initial clinical evaluation measures, the robot-trained group demonstrated significantly greater motor improvement (higher mean interval change ± standard error measurement) than the control group on the Motor Status and Motor Power scores for shoulder and elbow (see Table 1). In fact, the robot-trained group improved twice as much as the control group in these measures. These gains were specific to motions of the shoulder and elbow, the focus of the robot training. There were no significant between-group differences in the mean change scores for wrist and hand function. Similar results were gathered in patients who have had a paralyzed upper extremity after stroke for at least one year [8-10] (See Table 1).

**Table 1:** Mean interval change in impairment and disability (significance p < 0.05).

Between Group Comparisons: Final Evaluation Minus Initial Evaluation	Robot Trained (N = 55)	Control (N = 41)	P-Value
Impairment Measures (±sem)			
Motor Power (MP)	4.1 ± 0.4	2.2 ± 0.3	<0.01
Motor Status shoulder/elbow (MS-se)	8.6 ± 0.8	3.8 ± 0.5	<0.01
Motor Status Wrist/Hand (MS/wh)	4.1 ± 1.1	2.6 ± 0.8	NS

### Description of Robots

#### Modularity and Integration Potential

MIT's experience with well over 250 stroke patients has reinforced the importance of one of our core design specifications: *Modularity*. From the outset we believed that modularity is essential to success in robotic therapy, particularly in extending the approach to patients suffering from distinct afflictions. Consider, for example, patients undergoing surgery at the wrist (e.g., Colles Fracture) who might not require a device that manipulates the payload of all the degrees-of-freedom (DOF) of the arm. Therapy for these patients requires only the wrist robot [11-13]. Conversely there will be patients for whom different modules must be coupled to deliver therapy and carry the payload of the human arm. Presently MIT has deployed four modules into the clinic (Burke Rehabilitation): a planar 2-dof active module; vertical 1-dof active module; wrist 3-dof active module; and 1-dof passive grasp module.

#### Features common to all modules

All of our robot modules are specifically designed and built for clinical rehabilitation applications. Unlike most industrial robots, they are configured for safe, stable, and compliant operation in close physical contact with humans. This is achieved using backdrivable hardware and impedance control, a key feature of the robot control system. Each active module can move, guide or perturb movements of a patient's limb and can record motions and mechanical quantities such as the position, velocity, and forces applied.

The most profound engineering challenge specific to this family of robots is achieving the dual goals of high force production capability and backdrivability. Each module must be capable of generating sufficient force to move a patient's limb, but it must also itself be easily movable by an elderly or frail patient. Backdrivability is essential in keeping the patient engaged in the task and in allowing him to observe his successful and unsuccessful attempts at motion. Backdrivable hardware also improves the performance of systems controlled by impedance controllers. Achieving backdrivability and high force production, together in a single machine, is often difficult and becomes more so when the robot geometry is more complex.

The robot control system is an impedance controller that modulates the way the robot reacts to mechanical perturbation from a patient or clinician and ensures a gentle compliant behavior. Impedance control refers to using a control system (actuators, sensors and computer) to impose a desired behavior at a specified port of interaction with a robot, in this case the attachment of the robot to the patient's hand. Conceived in the early 1980's by one of the co-authors [14], it has been applied successfully in numerous robot applications that involve human-motor interaction. Impedance control has been extensively adopted by other robotics researchers concerned with human-machine interaction. In rehabilitation robotics impedance control has been successfully implemented in MIT-MANUS since its clinical debut in 1994. For robots interacting with the human, the most important feature of the controller is that its stability is extremely robust to the uncertainties due to physical contact [14,15]. The stability of most robot controllers is vulnerable when contacting objects with unknown dynamics. In contrast, dynamic interaction with highly variable and poorly characterized objects (to wit, neurologically impaired patients) will not de-stabilize the impedance controller above; even inadvertent contact with points other than the robot end-effector will not de-stabilize the controller. This is essential for safe operation in a clinical context.

#### Planar 2-dof robot MIT-MANUS

The MIT-MANUS project was initiated in 1989 with support from the National Science Foundation. MIT-MANUS has been in daily operation since 1994 delivering therapy to stroke patients at the Burke Rehabilitation Hospital. This robot has been extensively described in the literature [4] (See Figure 1). MIT-MANUS is a planar module which provides two translational degrees-of-freedom for elbow and forearm motion. The 2-dof module is portable (390 N) and consists of a direct-drive five bar-linkage SCARA (Selective Compliance Assembly Robot Arm). This configuration was selected because of its unique characteristics of low impedance on the horizontal plane and almost infinite impedance on the vertical axis. These allow a direct-drive backdrivable robot to easily carry the weight of the patient's arm. The mechanism is driven by brushless motors rated to 9.65 Nm of continuous stall torque with 16-bit virtual absolute encoders for position and velocity measurements (higher torques can be produced for limited periods of time). Redundant velocity sensing may be provided by DC-tachometers with a sensitivity of 1.8 V/rad/sec. A six degrees-of-freedom force sensor is mounted on the robot end-effector. The robot control architecture is implemented in a standard personal computer with 16-bit A/D and D/A I/O cards, as well as a DIO card with 32 digital lines. Besides its primary control function, this computer displays the task to both the operator and the subject or patient via dedicated monitors. Custom-made hand holders connect a patient's upper limb to the robot end-effector.

The selected design created a highly backdrivable robot capable of delivering therapy in a workspace of 15" by 18" with an end-point anisotropy of 2:1 ratio (2/3 < I < 4/3 Kg; 56.7 < static friction < 113.4 grams) and achievable impedances between 0 and 8 N/mm. Note that the static friction is significantly below the just noticeable difference (JNF) for force, which is 7% of the reference force. The robot maximum achievable impedance is above the human perception of 4.2 N/mm for a "virtual wall." The robot is capable of delivering forces up to 45 N although the robot target design aimed at a force of 28 N, which corresponds to the arm strength during elbow extension for a weak woman in seated position [16].

#### Vertical 1-dof Novel Robot

Following the successful clinical trials of MIT-MANUS, a 1-dof module to provide vertical motion and force was conceived and built. The primary goal of this module is to bring the benefits of planar therapy on MIT-MANUS to spatial arm movements, including movement against gravity. The module can be used independently or mounted to MIT-MANUS for movement in a limited spatial workspace. The module can permit free motion of the patient's arm, or can provide partial or full assistance or resistance as the patient moves against gravity. Because the vertical module moves with the endpoint of the planar module when the two are integrated, overall module mass is an important design concern in addition to on-axis mass and friction. Two embodiments are described below.

##### Screw-driven module

**Figure 2:**
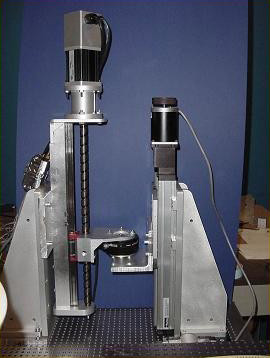
Constant-Velocity Friction Experiments (0.5 to 50 mm/sec). Photo shows alpha-prototype. The mean friction force was 20.075 ± 1.056 N.

One prototype of the 1-dof module was completed at MIT late 2000 and is shown alongside a test stand in Figure 2. A second clone was completed at MIT and deployed in the clinic (Burke Rehabilitation Hospital), where it is presently collecting pilot data with stroke patients. The module incorporates a custom-made "rollnut" and a custom-made screw with a linear guide system. Significant effort was engaged in the design of the screw transmission, which provides an efficient conversion of rotary to linear motion designed to eliminate nearly all-sliding friction in favor of rolling contact. Its low friction provides an intrinsically back-drivable design. The bracket mounted to the rollnut allows the attachment of different interfaces. Incorporated into the design are therapists' suggestions that functional reaching movements often occur in a range of motion close to shoulder scaption. Thus, the robotic therapy games that use the spatial robot focus on movements within the 45° to 65° range of shoulder abduction and from 30° to 90° of shoulder elevation or flexion. A Gripmate  is used to hold the patient's hand in place.

**Figure 3:**
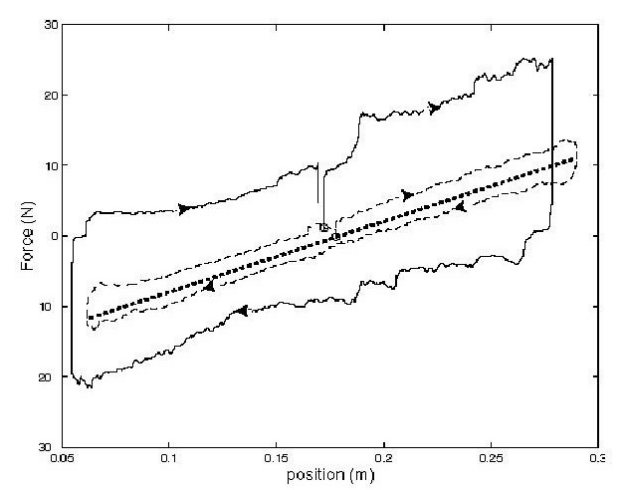
The graph shows force versus position with spring behavior commanded (heavy dot). PD controller alone (solid), PD controller with force feedback, *K*_*f *_= *5 *(dashed). Qualitatively, the roughly 3 N of friction force is almost imperceptible.

**Figure 4:**
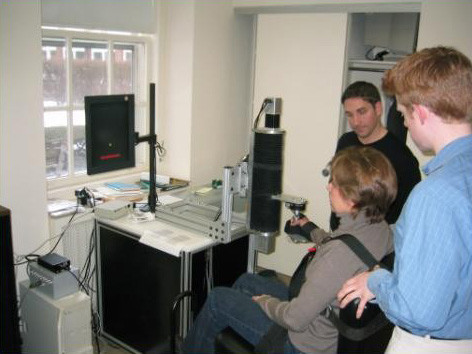
Graduates from Planar Robot Protocol Receiving Additional Vertical Anti-Gravity Training at the Burke Rehabilitation Hospital (White Plains, NY). The robot is sufficiently backdrivable to be lifted with the tip of the little finger.

**Figure 5:**
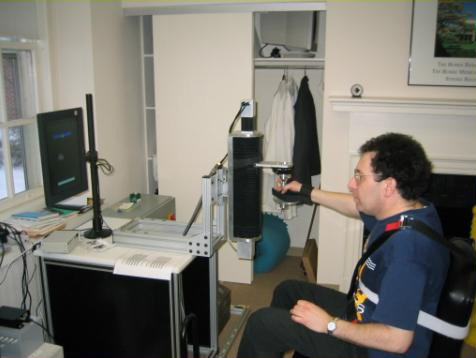
Graduates from Planar Robot Protocol Receiving Additional Vertical Anti-Gravity Training at the Burke Rehabilitation Hospital (White Plains, NY). The robot is sufficiently backdrivable to be lifted with the tip of the little finger.

This prototype has been fully characterized at MIT [17,18] (Figures 4 and 5). In comparison to MIT-MANUS, the vertical module has a greater effective endpoint mass and friction, though the resulting system is still back-drivable. In order to partially compensate for this increased impedance, force-feedback is incorporated into the impedance controller, resulting in a substantial reduction in friction, down to approximately 3 N, and mass, to approximately 1 kg. This improvement is illustrated in Figure 3. The module is capable of providing well over the force specification of 65 N in the upward direction (20 N estimate of patient's arm weigh) and 45 N in the downward direction, and can achieve stiffness in excess of 10 N/mm, far greater than the values generally used for therapy.

##### Linear direct-drive module

The screw-driven prototype has proven very successful both in standalone operation and mounted at the end of the planar module enabling spatial movement therapy in the clinic with compliant and stable behavior. However recent changes in linear motor technology have created the potential to achieve similar outcomes with effective vertical endpoint inertia comparable to the planar MIT-MANUS and much lower friction, without the need for force feedback control. The main change is complete enclosure of the magnets within the motor forcer. While this does not increase the magnetic field strength, it dramatically increases the line integral and concatenates magnetic lines.

**Figure 6:**
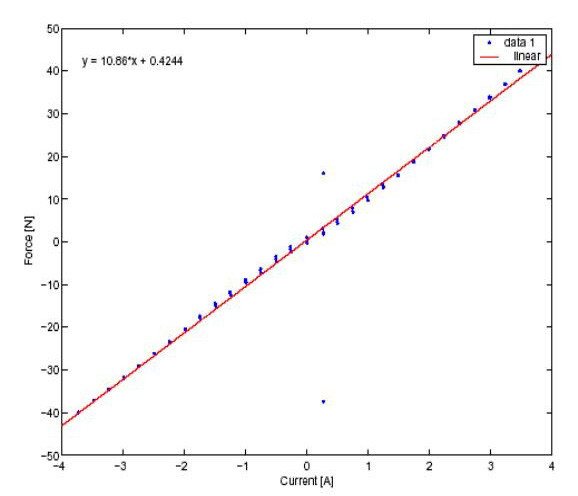
Characterization of TB2504. Plot shows the force versus current curve.

**Figure 7:**
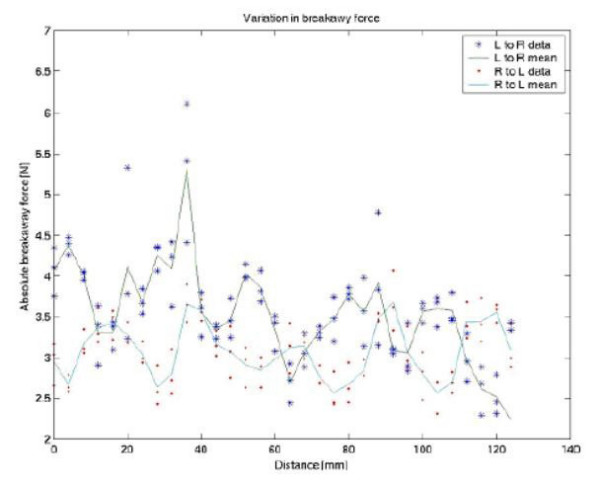
Characterization of TB2504. Plot shows the static friction and cogging.

**Figure 8:**
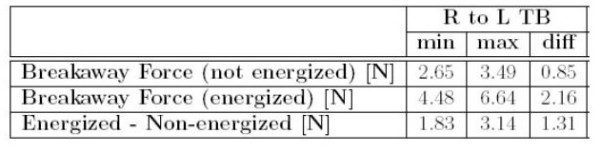
Characterization of TB2504.

**Figure 9:**
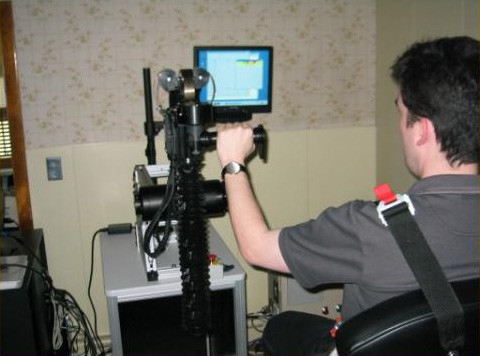
Vertical 1-dof Module Using Electrical Linear Technology. This commercial version of MIT's module can be operated in standalone fashion or integrated to the planar MIT-MANUS to allow spatial movements. Note that in the standalone fashion it can be operated at any angle to the horizontal and vertical planes with adjustable handle positions.

**Figure 10:**
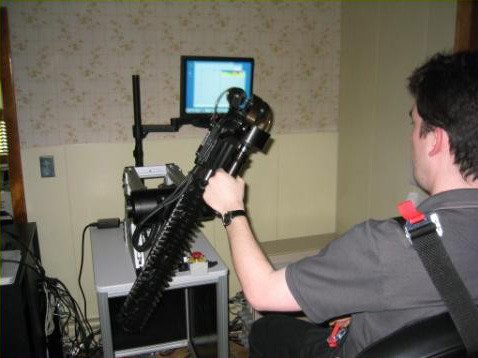
Vertical 1-dof Module Using Electrical Linear Technology. This commercial version of MIT's module can be operated in standalone fashion or integrated to the planar MIT-MANUS to allow spatial movements. Note that in the standalone fashion it can be operated at any angle to the horizontal and vertical planes with adjustable handle positions.

The practical advantages of converting the spatial system to direct-drive linear motors would be a significant reduction of friction and elimination of backlash. This simplification would also carry through to the control system and controller, as well as affording a reduction in the system's overall dimensions and weight. To determine if the expected friction levels are realistic, we tested Copley Control ThrustTube TB2504. Figures 6 ,7, 8 shows our experimental results characterizing the static friction for the TB series and the force vs current relationship. Figures 9 and 10 shows the commercial implementation of the novel module (Interactive Motion Technologies, Inc., Cambridge, MA). The novel module allows 19.4" of linear range of motion and it is capable of moving the desired target maximum endpoint force of 65 N upward and 45 N downward. This new module achieves significant reductions in friction and inertia to 25% and 76% of the lead-screw prototype.

#### Pilot Clinical Trials with the Anti-gravity Module

To test the novel vertical module we conducted a pilot study to analyze whether additional anti-gravity training further improves motor outcomes for "graduates" of the planar robot-assisted protocol.

##### In-/Exclusion Criteria

Outpatients were included in the study if they met the following criteria: a) first single focal unilateral lesion with diagnosis verified by brain imaging (MRI or CT scans) that occurred at least 6 months prior; b) cognitive function sufficient to understand the experiments and follow instructions (Mini-Mental Status Score of 22 and higher or interview for aphasic subjects); c) Motor Power score ≥1/5 or ≤3/5 (neither hemiplegic nor fully recovered motor function in the 14 muscles of the shoulder and elbow); d) informed written consent to participate in the study. Patients were excluded from the study if they have a fixed contracture deformity in the affected limb that limited pain-free range of motion. We have found severe tendon contractures around the rotator cuff particularly, in patients with complete hemiplegia for longer than 6 months after stroke. It is reasonable to expect that robotic training for the upper limb would not have an impact on a fixed contracture deformity. Trials commenced only after baseline assessment across three consecutive evaluations, 2 weeks apart, shows a stable condition in three motor impairment scales (F-M, MSS, MP). Our rationale for administering multiple baseline evaluations is based on an interesting "Hawthorne effect" that we observed in previous subjects [19,20]. Between first and second pre-treatment evaluations, some subjects have shown a remarkable improvement in clinical impairment scores. We speculate that the anticipated participation in a research study may contribute to a significant change in life routines.

##### Demographics

Ten (10) community dwelling volunteers who have suffered a single stroke at least 6 months prior to enrollment were enrolled in the pilot protocol. The mean group age was 62 ± 4.3 years old (mean ± sem) with the onset of the stroke occurring 50 ± 8.9 months (mean ± sem) prior to enrollment. Table 2 summarizes admission status of volunteers (See Table 2).

**Table 2:** Data on the Ten (10) Community Dwelling Stroke Volunteers

Age	Handed	Lesion foci	Lesion side	Months stroke	Fugl-Meyer adm (/66)
59	Left	AVM hem.	Left	16.5	11
53	Ambi	Intracerebral bleed	Left	88.5	18
44	Right	Carotid dissection	Left	36	11
63	Right	Cerebral embolism, subcortical	Left	58	9
82	Right	Cerebral embolism, subcortical	Left	69	9
72	Right	Carotid endarectomy	Right	24	24
41	Right	cortical/subcortical and basal ganglia stroke	Right	96	17
72	Right	cortical/subcortical stroke	Right	47.5	9
57	Right	Cerebral embolism, subcortical	Right	48	30
77	Right	cerebral embolism, mixed	Right	16	34

##### Description of Protocol

Patients were trained during the initial six-weeks with the planar module (i.e., training limited to horizontal movements with gravity compensation as in past studies). This training was followed by six-weeks of robotic therapy that focused on performing vertical arm movements against gravity. The 12-week protocol includes three one-hour robot therapy sessions per week (total 36 robot treatment sessions) (Figure 9).

**Figure 11:**
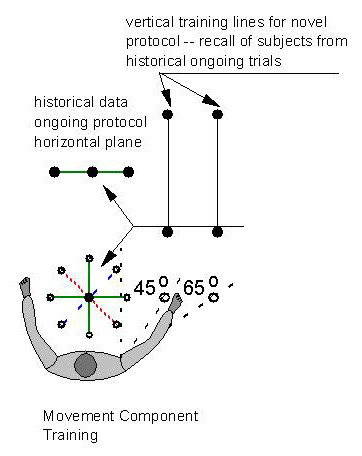
Movement Component Training. The circular display in front of the subject represents the workspace of the planar 6-weeks trial. The component training added 6 additional weeks training movements over two vertical lines.

For shoulder-and-elbow planar therapy, the center of the workspace was located in front of the subject at the body midline with the shoulder elevation at 30° with the elbow slighted flexed. The point-to-point movements started at the workspace center and extended in eight different directions of the compass (Figure 11). A one-hour session included two batches of 20 repetitions of point-to-point movements. The protocol incorporated a novel performance-based adaptive algorithm [21], which encouraged subjects to initiate movement with their hemiparetic arm.

Just as in the planar study [10], the anti-gravity robotic protocols consisted of visually evoked and visually guided point-to-point movements to different targets (along two vertical lines) with some robotic therapy games providing assistance and others visual feedback only. The protocol incorporated therapists' suggestions: a) robot therapy should focus on encouraging subjects to initiate movement against gravity with their hemiparetic arm beginning in a position of slight shoulder flexion (elevation) and scaption; b) functional reaching movements often occur in a range of motion close to shoulder scaption; c) no support should be provided at the elbow; and d) the visual display should be kept simple, since more complex displays proved to be difficult for our historical pool of stroke survivors to follow. Thus the robotic therapy protocols with the spatial robot focused on movements within the 45° to 65° range of shoulder abduction and between 30° to 90° of shoulder elevation or flexion. This sector is considered "safe" for the shoulder joint because it prevents gleno-humeral impingement that may occur when attempting to elevate the paretic limb to higher levels of shoulder elevation; The one-hour session included three batches of 20 repetitions of point-to-point movements. The first batch only provided visual feedback to a repetitive sequence of targets, while the second and third batches assisted the subject if needed to reach the target.

#### Clinical Assessment Scales

In this pilot, standard clinical evaluations included the upper extremity sub-test of the Fugl-Meyer Assessment (FM, maximum score = 66) from which we derived a Fugl-Meyer score for shoulder/elbow coordination (FM-SE, 42 out of 66); a more comprehensive evaluation of motor power or strength in 14 different muscle groups of the shoulder and elbow, using the MRC Motor Power score (MP, out of 70); and the Motor Status Score which is divided into two subscales, one for shoulder and elbow movements (MS-SE, maximum score = 40), and a second for wrist and hand abilities (MS-WH, maximum score = 42) [22,3,24,6,7]. The Modified Ashworth Scale (Bohannon, 1987) provides a measure of tone. The Fugl-Meyer test is a widely accepted measure of impairment in sensorimotor and functional grasp abilities [24]. To complement the Fugl-Meyer, we developed the Motor Status to further quantify discrete and functional movements in the upper limb. The MS-SE and MS-WH scales expand the F-M and have met the standards for inter rater reliability, significant intra-class correlation coefficients and internal item consistency [23,25].

## Results

We have completed the study with ten (10) outpatients. Nine (9) outpatients who completed the planar training protocol at the Burke Rehabilitation Hospital were enrolled for an additional six-weeks with training three sessions per week on the vertical module. One (1) naïve outpatient completed only the six-week training on the vertical module (no previous exposure to the planar training). Table 3 and 4 shows the results for the shoulder-and-elbow subcomponent of the upper extremity Fugl-Meyer scale, the Motor Status Score, Motor Power, and Modified Ashworth scale.

**Table 3:** Anti-Gravity Vertical Module Pilot Study. Results from nine (9) outpatients that continued for an additional 6 weeks of training in the vertical module robotic unit. Statistical tests showed that outcomes at discharge from planar robot protocol were distinct from admission (B vs. A), and there was a trend favoring further improvement when comparing discharge from anti-gravity protocol with discharge from the planar protocol (C vs. B). Our protocol was safe and did not increase tone.

Timeline N = 9	A – Admission	B – Discharge from planar robot protocol	C – Discharge from anti-gravity protocol
F-M s/e (/42)	12.7 ± 1.6	14.8 ± 2.0 (p = 0.03, S)	17.0 ± 1.9 (p = 0.19, NS)
MSS s/e (/40)	18.1 ± 1.9	19.9 ± 2.0 (p = 0.01, S)	21.5 ± 1.8 (p = 0.29, NS)
MP	26.5 ± 3.5	33.3 ± 3.6 (p < 0.01, S)	38.8 ± 2.4 (p = 0.07, NS)
Ashworth	8.0 ± 1.4	4.9 ± 0.99 (p < 0.03, S)	4.4 ± 1.01 (p = 0.67, NS)

**Table 4:** Anti-Gravity Vertical Module Pilot Study. Results from one naive (1) outpatient that trained for 6 weeks in the vertical module robotic unit (no prior robot exposure).

Timeline N = 1	B – Admission to anti-gravity protocol	C – Discharge from anti-gravity protocol
F-M s/e (/42)	24.0	27.0
MSS s/e (/40)	21.8	31.0
MP	35.0	43.0
Ashworth	4	1

Results of this pilot suggested that the vertical protocol was safe and well tolerated by the patients with no patient presenting any adverse effect (e.g., shoulder pain). Furthermore, pilot results suggested an additional reduction in shoulder-elbow impairment following the anti-gravity vertical training. In fact, for these 9 patients the reduction in impairment during the vertical training phase was comparable to the reduction during the planar phase (5.2% for the vertical training vs 5.0% for the planar training of the shoulder-elbow subcomponent of the Fugl-Meyer scale – albeit without achieving statistical significance). As mentioned earlier, we reliably incorporated therapists' feedback during the design phase of the protocol and have not observed increases in muscle tone or spasticity, as indicated by the Modified Ashworth scale, during the anti-gravity training protocol (actually the trend in 8 out of 9 patients is towards a decrease in tone). Note also that consistent with our past experience with the persons with chronic strokes, there was a statistically significant reduction in tone measurement from admission to discharge of performance-based planar robot therapy.

While the benefits from the additional anti-gravity therapy did not achieve statistical significance, this is likely due to the small sample size (9 outpatients). We anticipate that a modest increase of sample size will demonstrate statistical improvement for the shoulder and elbow of the anti-gravity training across the three clinical scales and we plan to commence trials shortly (See Table 3 and Table 4).

## Conclusions

Our clinical experiments have focused on a fundamental question of whether task specific robotic training influences brain recovery. To date several studies demonstrate that in mature and damaged nervous systems, nurture indeed has an effect on nature. The improved recovery is most pronounced in the trained limb segments (i.e. shoulder and elbow). We have now embarked on experiments that will test whether we can continue to influence recovery, long after the acute insult, with a novel class of robotic devices.

It is broadly accepted that outcome measures do not significantly change in persons with chronic motor impairments more than six months from stroke onset. However, recent task specific training programs have resulted in improved motor performance in persons with chronic stroke. For example, our trials with persons with chronic stroke-related impairments showed that planar robotic training contributed to statistically significant improvements in motor abilities [8-10]. If so, further robot training of stroke survivors who were enrolled in the planar robot trials might result in additional performance improvements when a distinct training protocol is provided.

Our pilot results and novel robotic modules provide a proof of concept that supports our engineering efforts, as well as further clinical trials to test efficacy. We found that the protocol was safe and well tolerated by the patients with no patient presenting any adverse effect (e.g., shoulder pain). Furthermore, the pilot results suggested an additional reduction in shoulder-elbow impairment following the anti-gravity vertical training without detrimental changes in muscle tone or spasticity. Therefore we plan to investigate in detail the effect of training each movement component in isolation versus integrated spatial movement, and study its impact on disability. We expect that this will bring us closer to our ultimate goal, efficient delivery of optimal therapy, personalized to serve the individual's needs.
